# Re-inspiration of CO_2 _from ventilator circuit: effects of circuit flushing and aspiration of dead space up to high respiratory rate

**DOI:** 10.1186/cc8986

**Published:** 2010-04-26

**Authors:** Edoardo De Robertis, Leif Uttman, Björn Jonson

**Affiliations:** 1 Department of Clinical Physiology, Lund University and Lund University Hospital, S-221 85, Lund, Sweden; 2 Department of Surgical, Anaesthesiological, and Intensive Care Medicine Sciences, University of Napoli Federico II, Via S. Pansini 5, Naples, 80131, Italy

## Abstract

**Introduction:**

Dead space negatively influences carbon dioxide (CO_2_) elimination, particularly at high respiratory rates (RR) used at low tidal volume ventilation in acute respiratory distress syndrome (ARDS). Aspiration of dead space (ASPIDS), a known method for dead space reduction, comprises two mechanisms activated during late expiration: aspiration of gas from the tip of the tracheal tube and gas injection through the inspiratory line - circuit flushing. The objective was to study the efficiency of circuit flushing alone and of ASPIDS at wide combinations of RR and tidal volume (V_T_) in anaesthetized pigs. The hypothesis was tested that circuit flushing and ASPIDS are particularly efficient at high RR.

**Methods:**

In Part 1 of the study, RR and V_T _were, with a computer-controlled ventilator, modified for one breath at a time without changing minute ventilation. Proximal dead space in a y-piece and ventilator tubing (VD_aw, prox_) was measured. In part two, changes in CO_2 _partial pressure (PaCO_2_) during prolonged periods of circuit flushing and ASPIDS were studied at RR 20, 40 and 60 minutes^-1^.

**Results:**

In Part 1, VD_aw, prox _was 7.6 ± 0.5% of V_T _at RR 10 minutes^-1 ^and 16 ± 2.5% at RR 60 minutes^-1^. In Part 2, circuit flushing reduced PaCO_2 _by 20% at RR 40 minutes^-1 ^and by 26% at RR 60 minutes^-1^. ASPIDS reduced PaCO_2 _by 33% at RR 40 minutes^-1 ^and by 41% at RR 60 minutes^-1^.

**Conclusions:**

At high RR, re-breathing of CO_2 _from the y-piece and tubing becomes important. Circuit flushing and ASPIDS, which significantly reduce tubing dead space and PaCO_2_, merit further clinical studies.

## Introduction

In acute respiratory distress syndrome, severe obstructive lung disease, and at increased intracranial pressure it may be important to maintain adequate CO_2 _exchange at low tidal volume ventilation (LTVV). LTVV will otherwise lead to respiratory acidosis. To uphold CO_2 _elimination, increased respiratory rate (RR) may then be applied [1]. At high RR, when dead space as a fraction of tidal volume increases, dead space reduction may be called for. A first step is to reduce the volume of connectors and humidifiers. A further step may be expiratory flushing of airways, later denoted tracheal gas insufflation (TGI) [2,3]. TGI is associated with problems related to humidification of the injected gas and of local effects of the jet stream at the tip of the tracheal tube. TGI will also disturb monitoring of ventilation. Therefore, a new technique, aspiration of dead space (ASPIDS) was developed and tested [4-6]. ASPIDS comprises two mechanisms, which are simultaneously activated late during expiration. One is *aspiration *of gas from the tip of the tracheal tube that is performed through a special lumen of the tracheal tube or through a catheter ending close to the tip of the tracheal tube. The other mechanism is gas injection through the inspiratory line, Circuit Flushing. Circuit Flushing compensates for the volume of aspirated gas and fills the inspiratory system with fresh gas. Before the ensuing inspiration, ASPIDS brings the interface between expired gas and fresh gas down to the tip of the tracheal tube.

After an ordinary expiration without ASPIDS or Circuit Flushing, CO_2 _is present at the start of inspiration in the Y-piece, in adjacent parts of the inspiratory tube and also in the expiratory tube. A volume of CO_2 _representing about 20 to 24 ml of alveolar gas is re-inspired from that zone during the inspiration [7,8]. It was reasoned that Circuit Flushing alone might clear this volume of CO_2_, thereby reducing dead space.

No systematic study has previously been performed to analyze how a wide range of RR and tidal volume (V_T_) combinations affects re-inspiration of dead space gas from the Y-piece and adjacent tubing. To what extent Circuit Flushing in itself contributes to the effects of ASPIDS at different RR and V_T _has not been studied. The objective of this study was to quantify re-inspiration from the Y-piece and adjacent parts of tubing at ordinary and increased RR and to examine the extent at which Circuit Flushing alone explains positive effects of ASPIDS at different combinations of RR and V_T_. The hypothesis was tested that ASPIDS and Circuit Flushing are particularly efficient at high RR.

## Materials and methods

The Ethics Board of Animal Research of Lund University approved the study. Five pigs of Swedish native breed weighing 19 to 23 kg were premedicated with xylazine (2 mg·kg^-1^), ketamine (15 mg·kg^1^) and atropine (0.5 mg). Anaesthesia was maintained by continuous intravenous infusion of fentanyl (60 μg·kg^-1^·h^-1^), midazolam (0.7 mg·kg^-1^·h^-1^), and ketamine (7 mg·kg^-1^·h^-1^). Paralysis was avoided to allow judgement of anaesthesia depth during the experiments. However, no muscular movements were observed. Initially the animals were hydrated with 1,000 ml Ringer-acetate (600 ml·h^-1^) followed by dextran at 200 ml·h^-1^. A femoral artery catheter was used for blood gas sampling (Radiometer ABL725, Copenhagen, Denmark) and blood pressure monitoring (HP 78353A). Mean arterial pressure (MAP) and pulse rate (HR) were monitored. Body temperature was maintained constant.

The animals were intubated with a 7.0 mm internal diameter tracheal tube connected to a ventilator (Servo Ventilator 900C, Siemens-Elema AB, Solna, Sweden). To minimize circuit dead space, the Y-piece was directly connected to the tracheal tube without swivel adaptor or humidifier. Ventilation was volume-controlled with square inspiratory flow pattern. At baseline, RR was 20 minutes^-1^, inspiratory time 33%, postinspiratory pause 5% and positive end-expiratory pressure (PEEP) 4 cmH_2_O. Below, RR is denoted RRnn, in which nn implies rate in minutes^-1^. The baseline minute ventilation (MV) was adjusted to achieve PaCO_2 _of 5 to 5.5 kPa. A mainstream CO_2 _analyser (CO_2 _Analyzer 930, Siemens-Elema, Solna, Sweden) was used to measure airway partial pressure of CO_2 _at the proximal end of the tracheal tube (PawCO_2_). The ventilator/computer system used for data recording and computer control of the ventilator has been described [9,10]. Signals from the ventilator and the CO_2 _analyzer representing flow rate, airway pressure and PawCO_2 _were sampled at 100 Hz. Compliance of the tracheal tube and ventilator tubing was measured in vitro. The system was tested for leakage. The animals were killed by an overdose of potassium chloride at the end of the experiment. There were no dropouts.

### ASPIDS circuit

**Figure 1:**
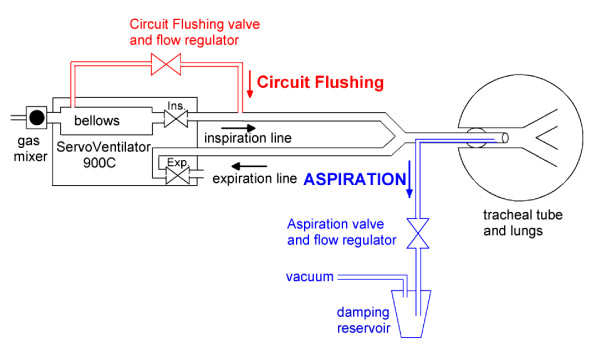
**ASPIDS system**. The Servo Ventilator 900C complemented by a system for Circuit Flushing (in red) and a system for aspiration of gas from the tip of the tracheal tube (in blue). During Circuit Flushing only the red valve is opening during the last third of the expiration period. During ASPIDS both red and blue valves are opening. A development suggested in the Discussion is to program the regular inspiratory flow regulating valve (Ins.) to perform Circuit Flushing without any extra tube or other hardware.

The ASPIDS system, comprising the Servo Ventilator 900C, an electronic control unit, and two valves, has been described in detail [5]. One valve, used for Aspiration, connects a vacuum source to the aspiration catheter (ID 2.5 mm, OD 2.9 mm) ending 2 cm proximal to the tip of the tracheal tube. The other, used for Circuit Flushing, connects the bellow of the ventilator to the inspiratory line, Figure 1. Aspiration and/or Circuit Flushing were performed over the last 30% of expiration time. Flow rate and volume for Aspiration and Circuit Flushing were adjustable. Aspiration volume was 5 to 10 ml lower than Circuit Flushing volume. The ASPIDS period is short at high RR. Therefore, Circuit Flushing flow rate, being 0.22 L·sec^-1 ^at RR20 and 40, was increased to about 0.35 L·sec^-1 ^at RR60 to assure that flushing and aspiration volumes were not less than 60 ml and sufficient to clear the tracheal tube.

### Protocol

After animal preparation, a stabilisation period at basal ventilation was allowed for 60 minutes to establish a steady state. The protocol had two parts. The experiment was performed with a previously described computer controlled ventilator [9].

Part 1: After the stabilisation period, the effect of different combinations of RR and V_T _on dead space from the Y-piece and adjacent parts of the ventilator tubing was analyzed without using ASPIDS or Circuit Flushing. At basal ventilation at RR20, single breaths were modified, with respect to RR and V_T_. Sequences of 10 breaths were recorded. The second and seventh breaths were modified under computer control. Between modified breaths were ordinary breaths. The combination of RR and V_T _was for each modified breath such that minute ventilation remained unchanged. For modified breaths RR was 10, 30, 40, 50 or 60 minutes^-1 ^while V_T _was inversely modified. In randomized order, each RR-V_T _combination was recorded three times. Other parameters like PEEP were constant. The computer was programmed to modify single breaths at a time to allow comparisons with ordinary breaths within the same recording as in previous studies [10-12].

**Figure 2:**
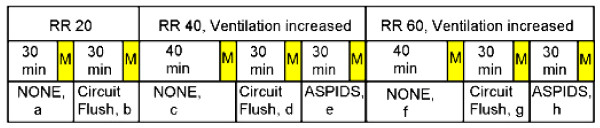
**Protocol for Part 2**. At RR 20, 40 and 60 equilibration time preceded measurements denoted M, during ordinary ventilation (NONE), Circuit Flushing (Circuit Flush) and complete ASPIDS. Letters **a-h **correspond to instances described in the text.

In Part 2 measurements at steady state were made of ventilation parameters, blood gases and haemodynamics at basal ventilation, at Circuit Flushing alone and at complete ASPIDS at various combinations of RR and V_T_. PaCO_2 _was measured every 10 minutes. Dead space can not be measured during Circuit Flushing and ASPIDS. The following scheme, also depicted in Figure 2, was followed:

a. Basal ventilation at RR20. Measurements after 30 minutes.

b. Circuit Flushing started at RR20. Measurements after 30 minutes.

c. Circuit Flushing stopped and RR increased to 40 minutes^-1^. Minute ventilation increased to maintain a stable CO_2 _elimination rate as read from the CO_2 _analyzer. Measurements after 40 minutes.

d. Without changing RR, Circuit Flushing started. Measurements after 30 minutes.

e. Aspiration started for complete ASPIDS. Measurements after 30 minutes.

f. Circuit Flushing and aspiration stopped and RR increased to 60 minutes^-1^. Minute ventilation increased to maintain stable CO_2 _elimination rate. Measurements after 40 minutes.

g. Procedure d repeated at RR60.

h. Procedure e repeated at RR60.

### Data analysis

**Figure 3:**
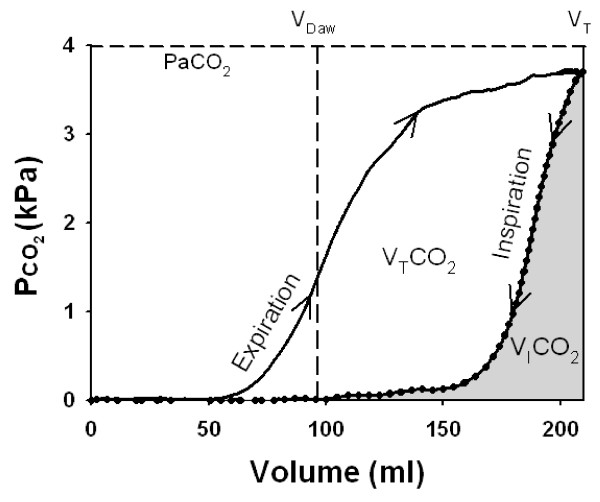
**SBT-CO_2 _of a representative animal**. Partial pressure of CO_2 _in expired gas (solid line) and inspired gas (dotted line) plotted against volume so as to create a loop. The area within the loop corresponds to tidal elimination of CO_2 _(V_T_CO_2_). The area below the inspiratory limb (grey) corresponds to re-inspired volume of CO_2 _proximal of the CO_2 _sensor (V_I_CO_2_). Airway dead space distal to the CO_2 _sensor (V_Daw_) is indicated (vertical interrupted line).

Sampled data of flow rate, airway pressure and PawCO_2 _were transferred to a spreadsheet (Excel 2003, Microsoft, Redmond, WA, USA). The single-breath test for CO_2 _was analyzed according to principles described by Beydon *et al *[7]. The volume of CO_2 _eliminated per breath (V_T_CO_2_) corresponds to the area within the loop, Figure 3. The volume of CO_2 _re-inspired from the Y-piece and tubing per breath (V_I_CO_2_) is reflected by the area to the right of the loop. Dead space proximal to the CO_2 _sensor caused by V_I_CO_2_(VD_aw, prox_) was calculated:(1)

Pe'CO_2 _is the end-tidal CO_2 _and Pbar barometric pressure. VD_aw, prox _in % of V_T _is denoted VD_aw, prox_%.

Airway dead space distal to the CO_2 _sensor (V_Daw_) was determined according to an algorithm of Wolff and Brunner [13] modified to correct for a sloping alveolar plateau [10].

### Statistical analysis

All data are expressed as mean ± standard deviation (SD). Student's paired two-tailed *t*-test was used. Linear and logarithmic regressions were applied. *P *values less than 0.05 were considered significant.

## Results

During the whole procedure, all animals remained stable with respect to oxygenation and arterial blood pressure. Heart rate showed a trend to increase from on average 74 ± 20 to 94 ± 22 minutes^-1 ^(Table 1).

**Table 1:** Effects of Circuit Flushing and ASPIDS at increasing respiratory rate

RESPIRATORY RATE		MV, **Lit**.	V_T_, ml	Pplat, cmH_2_O	Cst, ml/cmH_2_0	V'CO_2_ ml/min	PaCO_2_, kPa	pH	PaO_2_, kPa	HR, b/min	MAP, mmHg

**RR20**	**Baseline**	4.1 ± 0.6	208 ± 31	14 ± 2.6	20 ± 3	133 ± 10	5.3 ± 0.2	7.47 ± 0.04	12.3 ± 1.2	74 ± 20	84 ± 17

	**After 30 minutes of Circuit Flushing**	4.1 ± 0.6	208 ± 31	14 ± 3.5			4.7 ± 0.4 *	7.51 ± 0.04 *	12.6 ± 1.2	78 ± 20 **	83 ± 15


**RR40**	**Baseline**	4.9 ± 0.5	130 ± 13	13 ± 3.7	15 ± 4	142 ± 20	5.9 ± 0.4 #	7.43 ± 0.04	10.7 ± 0.9	79 ± 15	81 ± 12

	**After 30 minutes of Circuit Flushing**	4.9 ± 0.5	130 ± 13	14 ± 3.5 **			4.7 ± 0.3 **	7.51 ± 0.04 **	12 ± 1.5	81 ± 13	80 ± 9

	**After 30 minutes of Circuit Flushing + ASPIDS**	4.9 ± 0.5	130 ± 13	13 ± 3.6			3.9 ± 0.2 *	7.58 ± 0.05 **	12 ± 1.8	84 ± 11	79 ± 10


**RR60**	**Baseline**	5.9 ± 0.5	101 ± 9.5	13 ± 2.2	12 ± 2	136 ± 16	6.3 ± 0.4 #	7.40 ± 0.05	9.6 ± 1.3	84 ± 9	81 ± 9

	**After 30 minutes of Circuit Flushing**	5.9 ± 0.5	101 ± 9.5	14 ± 2			4.6 ± 0.6 **	7.51 ± 0.07 *	11.5 ± 2 *	91 ± 20	83 ± 9

	**After 30 minutes of Circuit Flushing + ASPIDS**	5.9 ± 0.5	101 ± 9.5	12 ± 2 **			3.7 ± 0.5 **	7.59 ± 0.07 **	11.2 ± 2.8	94 ± 22	79 ± 11

* *P *< 0.01; ** *P *< 0.001 (comparison were made to the preceding value). # *P *<0.05 (comparison between baseline at RR40 and 60 vs baseline at RR20, and between baseline at RR60 vs baseline at RR40). ASPIDS, aspiration of dead space; Cst, static compliance; HR, heart rate; MAP, mean arterial pressure; MV, minute ventilation; PaCO_2, _carbon dioxide arterial partial pressure; PaO_2, _oxygen arterial partial pressure; Pplat, plateau pressure; RR, respiratory rate; V_T, _tidal volume; V'CO_2, _carbon dioxide production.

### Part 1

**Figure 4:**
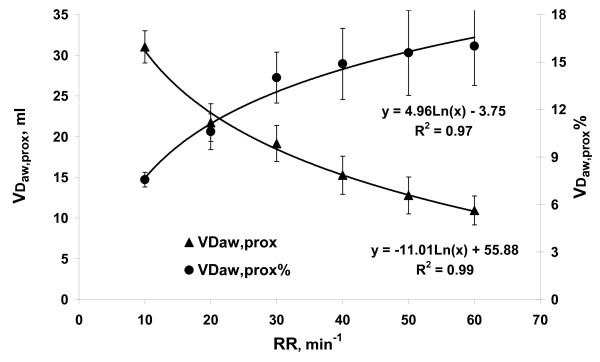
**Proximal airway dead space in ml (VD_aw, prox_), and in % of tidal volume (VD_aw, prox_%) related to respiratory rate (RR)**. Black lines represent the logarithmic fit.

At increasing RR, VD_aw, prox _decreased from 31 ± 2 ml at RR10 to 11 ± 2 ml at RR60 tightly according a logarithmic equation (Figure 4). VD_aw, prox _% was 7.6 ± 0.5% at RR10 and increased logarithmically to 16 ± 2.5% at RR60 (Figure 4). Peak expiratory flow decreased with RR according to the equation: y = - 0.33 Ln(RR) + 0.85, (R^2 ^= 0.99).

### Part 2

Table 1 shows the effects of Circuit Flushing and ASPIDS in comparison to basal ventilation at RR20 to 60. Minute ventilation and V_T _were maintained at all settings.

Compared to baseline ventilation, Circuit Flushing reduced PaCO_2 _by 10, 20 and 26% at RR20, RR40 and RR60, respectively. ASPIDS reduced PaCO_2 _by 33% at RR40 and 41% at RR60, Table 2. Accordingly, the reduction in PaCO_2 _achieved by Circuit Flushing alone was at RR40 60% of the total ASPIDS effect and 63% at RR60.

**Table 2:** Change in PaCO_2 _ in % of baseline value at each RR

RR, min^-1^	20	40	60
	**Change in PaCO_2_ in % of baseline value at each RR**		

**Circuit Flushing**	-10.3 ± 4, *P *= 0.005	-20 ± 3, *P *= 0.0002	-26 ± 7, *P *= 0.001

**ASPIDS**	-	-33 ± 5, *P *= 0.0004	-41 ± 6, *P *= 0.0002

ASPIDS, aspiration of dead space; PaCO_2, _carbon dioxide arterial partial pressure; RR, respiratory rate.

**Figure 5:**
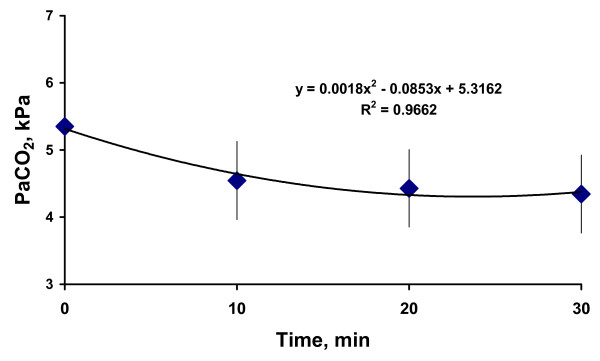
**Average of PaCO_2 _evolution during Circuit Flushing and ASPIDS periods**.

During Circuit Flushing and ASPIDS period PaCO_2 _decreased fast during the first 10 minutes and later at a slower rate in accordance with the equation: y = 0.0018x^2^-0.085x + 5.3 (R^2 ^= 0.97) (Figure 5).

## Discussion

The study was performed in healthy pigs to allow a detailed analysis over several hours without problems related to patient care and physiological stability. The study relates to events in ventilator tubing, y-piece and tracheal tube, which are relatively independent on the physiology of the subject studied. The principle results should be valid also in a clinical context. To what extent the dead space reduction achieved with ASPIDS and Circuit Flushing is of clinical value can only be judged from clinical studies.

In previous experiments with ASPIDS at health [4,6] and in animals and patients with acute respiratory failure [5,14] V_T _and airway pressures were reduced while normocapnia was maintained. The present study is the first in which ASPIDS and also Circuit Flushing was shown to modify PaCO_2_. This is also the first comprehensive analysis of how a wide range of V_T _- RR combinations affect airway dead space resulting from re-inspiration of CO_2 _from Y-piece and adjacent tubing. In confirmation of the hypothesis it was shown that ASPIDS and Circuit Flushing are particularly efficient at high RR. It was shown for the first time that Circuit Flushing significantly may enhance CO_2 _elimination and reduce PaCO_2 _through its effects on VD_aw, prox_. This aspect may be important for future development because Circuit Flushing can very easily be implemented as further discussed below.

In Part 1 it was shown that V_Daw, prox _in ml decreased at higher RR and correspondingly lower V_T_, in line with previous observations [10]. This reflects that V_Daw, prox _reflects admixture of CO_2 _to the inspiratory ventilator line during expiration and re-inspiration of CO_2 _from both ventilator lines during inspiration. These phenomena are related to diffusion, turbulence, Venturi, and Coandă effects around the Y-piece [7,8,10]. At higher RR, less time is available for these phenomena, while expiratory flow rate that promotes gas mixing in tubing around the y-piece is lower, as shown. Thereby, V_Daw, prox _becomes lower at high RR. However, V_Daw, prox_% increased two-fold over the interval RR10 to RR60 in spite of that V_Daw, prox _in ml fell to one third. These data (Figure 4) show that the importance of CO_2 _re-inspiration from ventilator lines and Y-pieces increases at a high RR which is essential for understanding the results in Part 2.

Fletcher *et al*. suggested the use of non-return valves in the Y-piece to avoid re-breathing [8]. Safety issues might be a reason why such valves have not been introduced. At present the need for ventilation at low V_T _and high RR asks for a safe solution of the significant re-breathing problem.

In Part 2, a period of 30 to 40 minutes was allowed for steady state establishment on the basis of previous data [15]. Longer periods would increase risks of significant changes in physiological status of the animals. Data in Figure 5 confirm that a steady state was achieved.

ASPIDS clears the tubing of CO_2 _down to the tip of the tracheal tube, while Circuit Flushing only clears tubes to and into the y-piece. Therefore, as expected, the effect on PaCO_2 _of ASPIDS was more important than that of Circuit Flushing. Still, the effect of Circuit Flushing was about 60% of the full ASPIDS effect. This reflects that the y-piece was connected directly to the tracheal tube, thereby minimising the apparatus dead space that is cleared of CO_2 _only by ASPIDS. While ASPIDS optimally reduces re-inspiration of CO_2 _from ventilator lines, Circuit Flushing is an easier technique to implement. No extra tube or channel is needed in the tracheal tube and no system for aspiration. As many modern ventilators have a computer controlled inspiratory pneumatic system, Circuit Flushing can be achieved by programming this system to perform Circuit Flushing without any extra tubes, valves or other hardware.

In a recent Editorial Frutos-Vivar *et al*. suggested that in ARDS 'the ideal ventilation would be that one that does not damage respiratory muscles or lung parenchyma' and 'that individual tailoring may be necessary' [16]. Lung parenchyma is damaged by barotrauma, related to high airway pressure, and by shearing forces at tidal lung collapse and re-opening. Limitation of airway pressure to prevent barotrauma while applying a PEEP high enough to keep the lung open, calls for low or even very low V_T_. One must consider that a particular dead space reduction allows more than an equal reduction in V_T_, because it also paves the way for an extra increase in RR and a secondary reduction in V_T_. This can be understood by considering a system in which dead space would approach zero. Then, V_T _can be reduced toward zero by approaching infinite RR. PEEP and peak pressure would be similar and lung protection from damaging forces could be truly optimized. The more efficient elimination of CO_2 _using Circuit Flushing and ASPIDS at RR40 and RR60 would in a clinical setting allow a significant reduction in V_T _and serve as one step in the direction of lung protection. It is realized that *tailoring *means much more. In an animal ARDS model, Uttman *et al*. recently studied how V_T _might be reduced by tailoring ventilation to actual lung mechanics and dead space [17]. V_T _could be modestly reduced from 7.2 to 6.6 ml/kg when RR was increased from 40 to 60 minutes^-1 ^and other ventilation parameters optimized. By using ASPIDS, V_T _could be further reduced to 4.0 ml/kg at RR of 80 minutes^-1^. It is realized that application of very high respiratory rates is associated with high requirements of tuning ventilation to circumstances. It is associated with significant difficulties with respect to monitoring. Dead space reduction is only a part of a complex strategy. With all respect for the difficulties, it is time to perform clinical studies in which true tailoring of ventilation to physiology is adapted to clinical circumstances and then to apply such techniques in controlled studies.

## Conclusions

In conclusion, re-breathing of CO_2 _rich gas present in the circuit line, although not clinically relevant at health and at low respiratory rates, should be considered when high frequencies are used. Circuit Flushing and ASPIDS were confirmed to be safe and efficient techniques to reduce tubing dead space, re-breathing of CO_2 _and, accordingly, PaCO_2_. Our results merit further studies in clinical settings and in different categories of critically ill patients.

## Key messages

• Re-breathing of CO_2_, although not clinically relevant at health and at low RR, should be considered at high RR.

• Minimizing circuit dead space, Circuit Flushing explains 60% of the full Aspiration of dead space.

• Circuit Flushing and Aspiration of dead space are safe and efficient techniques to reduce tubing dead space, re-breathing of CO_2 _and, PaCO_2_.

## Abbreviations

ASPIDS: aspiration of dead space; LTVV: low tidal volume ventilation; MV: minute ventilation; PawCO_2_: airway partial pressure of CO_2 _at the proximal end of the tracheal tube; PEEP: positive end-expiratory pressure; RR: respiratory rate; TGI: tracheal gas insufflation; VD_aw, prox_: proximal airway dead space; V_Daw_: airway dead space; V_I_CO_2_: CO_2 _re-inspired from Y-piece and tubing per breath; V_T_: tidal volume; V_T_CO_2_: volume of CO_2 _eliminated per breath.

## Competing interests

The authors declare that they have no competing interests.

## Authors' contributions

EDR designed the study, carried out the experiments, analysed row data and drafted the manuscript. LU carried out the experiments and analysed row data. BJ participated in the study design, coordinated the study, and helped to draft the manuscript. All authors read and approved the final manuscript.

## References

[B1] The Acute Respiratory Distress Syndrome Network Ventilation with lower tidal volumes as compared with traditional tidal volumes for acute lung injury and the acute respiratory distress syndrome N Engl J Med 2000 342 1301 1308 10.1056/NEJM200005043421801 10793162

[B2] Jonson B Similowski T Levy P Viires N Pariente R Expiratory flushing of airways: a method to reduce deadspace ventilation Eur Respir J 1990 3 1202 1205 2128626

[B3] Marini JJ Tracheal gas insufflation: a useful adjunct to ventilation? Thorax 1994 49 735 737 10.1136/thx.49.8.735 8091315 PMC475115

[B4] De Robertis E Sigurdsson S Drefeldt B Jonson B Aspiration of airway dead space. A new method to enhance CO2 elimination Am J Respir Crit Care Med 1999 159 728 732 10051243 10.1164/ajrccm.159.3.9712140

[B5] De Robertis E Servillo G Jonson B Tufano R Aspiration of dead space allows normocapnic ventilation at low tidal volumes in man Intensive Care Med 1999 25 674 679 10.1007/s001340050929 10470570

[B6] De Robertis E Servillo G Tufano R Jonson B Aspiration of dead space allows isocapnic low tidal volume ventilation in acute lung injury. Relationships to gas exchange and mechanics Intensive Care Med 2001 27 1496 1503 10.1007/s001340101046 11685343

[B7] Beydon L Uttman L Rawal R Jonson B Effects of positive end-expiratory pressure on dead space and its partitions in acute lung injury Intensive Care Med 2002 28 1239 1245 10.1007/s00134-002-1419-y 12209271

[B8] Fletcher R Werner O Nordstrom L Jonson B Sources of error and their correction in the measurement of carbon dioxide elimination using the Siemens-Elema CO_2 _Analyzer Br J Anaesth 1983 55 177 185 10.1093/bja/55.2.177 6219687

[B9] Svantesson C Drefeldt B Sigurdsson S Larsson A Brochard L Jonson B A single computer-controlled mechanical insufflation allows determination of the pressure-volume relationship of the respiratory system J Clin Monit Comput 1999 15 9 16 10.1023/A:1009916905078 12578056

[B10] Åström E Uttman L Niklason L Aboab J Brochard L Jonson B Pattern of inspiratory gas delivery affects CO_2 _elimination in health and after acute lung injury Intensive Care Med 2008 34 377 384 10.1007/s00134-007-0840-7 17763841

[B11] Devaquet J Jonson B Niklason L Si Larbi AG Uttman L Aboab J Brochard L Effects of inspiratory pause on CO_2 _elimination and arterial PCO_2 _in acute lung injury J Appl Physiol 2008 105 1944 1949 10.1152/japplphysiol.90682.2008 18801962 PMC2956750

[B12] Aboab J Niklason L Uttman L Kouatchet A Brochard L Jonson B CO_2 _elimination at varying inspiratory pause in acute lung injury Clin Physiol Funct Imaging 2007 27 2 6 10.1111/j.1475-097X.2007.00699.x 17204030

[B13] Wolff G Brunner JX Series dead space volume assessed as the mean value of a distribution function Int J Clin Monit Comput 1984 1 177 181 10.1007/BF01872769 6546139

[B14] Uhlig S Ranieri M Slutsky AS Biotrauma hypothesis of ventilator-induced lung injury Am J Respir Crit Care Med 2004 169 314 315 14718244 10.1164/ajrccm.169.2.950

[B15] Taskar V John J Larsson A Wetterberg T Jonson B Dynamics of carbon dioxide elimination following ventilator resetting Chest 1995 108 196 202 10.1378/chest.108.1.196 7606958

[B16] Frutos-Vivar F Ferguson ND Esteban A Mechanical ventilation: quo vadis? Intensive Care Med 2009 35 775 778 10.1007/s00134-009-1450-3 19288078

[B17] Uttman L Ögren H Niklason L Drefeldt B Jonson B Computer simulation allows goal-oriented mechanical ventilation in acute respiratory distress syndrome Crit Care 2007 11 R36 10.1186/cc5719 17352801 PMC2206452

